# The Definition and Delineation of the Target Area of Radiotherapy Based on the Recurrence Pattern of Glioblastoma After Temozolomide Chemoradiotherapy

**DOI:** 10.3389/fonc.2020.615368

**Published:** 2021-02-22

**Authors:** Lin Zheng, Zhi-Rui Zhou, QianQian Yu, Minghan Shi, Yang Yang, Xiaofeng Zhou, Chao Li, Qichun Wei

**Affiliations:** ^1^ Department of Radiation Oncology, The Second Affiliated Hospital, Zhejiang University School of Medicine, Hangzhou, China; ^2^ Department of Radiation Oncology, Taizhou Cancer Hospital, Wenling, China; ^3^ Radiation Oncology Center, Huashan Hospital, Shanghai Medical College, Fudan University, Shanghai, China; ^4^ Département de l’éducation aux adultes, Cégep Saint-Jean-sur-Richelieu, Brossard, QC, Canada

**Keywords:** glioblastoma, recurrence pattern, temozolomide, chemoradiotherapy, peritumoral edema

## Abstract

Radiotherapy is an important treatment for glioblastoma (GBM), but there is no consensus on the target delineation for GBM radiotherapy. The Radiation Therapy Oncology Group (RTOG) and European Organisation for Research and Treatment of Cancer (EORTC) each have their own rules. Our center adopted a target volume delineation plan based on our previous studies. This study focuses on the recurrence pattern of GBM patients whose target delineations did not intentionally include the T2/fluid-attenuated inversion recovery (FLAIR) hyperintensity area outside of the gross tumor volume (GTV). We prospectively collected 162 GBM cases and retrospectively analysed the clinical data and continuous dynamic magnetic resonance images (MRI) of 55 patients with recurrent GBM. All patients received concurrent radiotherapy and chemotherapy with temozolomide (TMZ). The GTV that we defined includes the postoperative T1-weighted MRI enhancement area and resection cavity. Clinical target volume 1 (CTV1) and CTV2 were defined as GTVs with 1 and 2 cm margins, respectively. Planning target volume 1 (PTV1) and PTV2 were defined as CTV1 and CTV2 plus a 3 mm margin with prescribed doses of 60 and 54 Gy, respectively. The first recurrent contrast-enhanced T1-weighted MRI was introduced into the Varian Eclipse radiotherapy planning system and fused with the original planning computed tomography (CT) images to determine the recurrence pattern. The median follow-up time was 15.8 months. The median overall survival (OS) and progression-free survival (PFS) were 17.7 and 7.0 months, respectively. Among the patients, 44 had central recurrences, two had in-field recurrences, one had marginal recurrence occurred, 11 had distant recurrences, and three had subependymal recurrences. Five patients had multiple recurrence patterns. Compared to the EORTC protocol, target delineation that excludes the adjacent T2/FLAIR hyperintensity area reduces the brain volume exposed to high-dose radiation (P = 0.000) without an increased risk of marginal recurrence. Therefore, it is worthwhile to conduct a clinical trial investigating the feasibility of intentionally not including the T2/FLAIR hyperintensity region outside of the GTV.

## Introduction

Glioblastoma (GBM) is the most common primary malignant central nervous system tumor with an incidence of approximately 3.20/100,000, accounting for 46.1% of all gliomas (1). The current standard treatment includes maximal safe resection of the tumor, followed by local radiotherapy and concurrent and adjuvant chemotherapy with temozolomide (TMZ) (2, 3). The prognosis of GBM patients is poor. Even with active standard treatment, the median overall survival (OS) is only 14.6 months (2). It seems inevitable for GBM to recur. At present, the GBM radiotherapy protocols of Radiation Therapy Oncology Group (RTOG) or European Organisation for Research and Treatment of Cancer (EORTC) are commonly used. The RTOG protocol is as follows (4): The first stage comprises 46 Gy in 23 fractions. Gross tumor volume 1 (GTV1) includes the postoperative T1-weighted magnetic resonance images (MRI) enhancement area, resection cavity, and abnormal signal area in the T2-weighted and fluid-attenuated inversion recovery (FLAIR) magnetic resonance images (MRI) phases. Clinical target volume 1 (CTV1) is defined as a 2 cm expansion from GTV1. If there is no edema around the tumor, 2.5 cm from GTV1 is added. Planning target volume 1 (PTV1) is expanded 0.3–0.5 cm from CTV1 and varies in different centers. The second stage is irradiation with 14 Gy in seven fractions. GTV2 includes the enhancing area of T1-weighted MRI and the resection cavity. CTV2 is expanded 2 cm from GTV2. PTV2 is the same range as in the first stage. The EORTC protocol is as follows (5): one target volume of 60 Gy in 30 fractions is prescribed. GTV includes the T1-weighted MRI enhancement area and resection cavity but does not intentionally include the peritumoral edema area. CTV is obtained by GTV extended 2 cm. PTV is treated with 0.3–0.5 cm expansion according to each center.

Thus, there is no consensus on the target delineation for GBM. The debate is over whether it is necessary to treat all possible tumor microinvasion locations. The theoretical basis for including peritumoral edema is that the edema area may be an infiltrative area (6, 7). The theoretical basis for excluding edema is that there is no significant difference in recurrence patterns either with or without edema. The target volume, including the edema area, increases the radiation volume of normal brain tissue and the risk of brain injury (8). To date, no agreement has been reached on whether the target volume of high-grade glioma should include edema.

Our center conducted a retrospective analysis of high-grade glioma treated before June 2014 and found that the incidence of tumor marginal recurrence did not increase in the target volume without intentionally including adjacent T2/FLAIR hyperintensity areas (9). Since then, we delineated the enhancing area of T1-weighted MRI and the resection cavity as GTV for GBM patients. For CTV, we did not intend to include all the adjacent T2/FLAIR hyperintensity areas, and only edema in the expansion area was included. A total of 162 cases of GBM were treated from January 1, 2015, to June 1, 2018. These cases were analysed and summarised, with emphasis on the recurrence pattern of the tumors, and the prognosis of the patients.

## Materials and Methods

### Patients

We prospectively collected the data of GBM patients admitted to our department from January 1, 2015, to June 1, 2018. Fifty-five patients had follow-up information. We performed a retrospective analysis of their clinical data and continuous dynamics MRI. All the selected patients had complete dynamic cranial MRI follow-up data for their tumor, which had been surgically resected and confirmed by pathology (GBM pathological diagnosis criteria were based on the fourth edition of the 2016 World Health Organization central nervous system tumor classification). Histopathological sections before 2016 were re-evaluated according to the latest diagnostic criteria and pathologically confirmed as GBM. All patients received radiotherapy and concurrent chemotherapy and adjuvant chemotherapy with TMZ. O^6^-methylglucamine-DNA methyltransferase (MGMT) promoter methylation status was tested using a methylation-specific polymerase chain reaction (PCR) method. Isocitrate dehydrogenase 1/2 (IDH1/2) mutations were determined by fluorescence *in situ* hybridization (FISH). Clinical, pathological, and imaging data were collected from the Picture Archiving and Communication Systems (PACS) and electronic medical record (EMR) system of the Second Affiliated Hospital of Medical College of Zhejiang University. The Ethics Committee of the Second Affiliated Hospital of Medical College of Zhejiang University approved the retrospective analysis of patient data.

### Treatment Details

Adjuvant radiotherapy started within 2–4 weeks of surgery. Radiotherapy planning was performed using the Varian Eclipse radiotherapy planning system. According to our radiotherapy plan, the standard prescription dose was 60 Gy (once a day, 2 Gy/Fx, Monday to Friday) for 6 weeks. GTV was defined as the enhanced area and resection cavity on contrast-enhanced T1-weighted MRI. CTV1 and CTV2 were expanded from the GTV by 1 and 2 cm, respectively. We used the following CTV delineation modification principle (5): 0 mm (bone window) from the skull boundary, 5 mm from the cerebellar tentorium or ventricle, and 0 mm from the brainstem, optic nerve, and optic chiasma. The adjacent T2/FLAIR hyperintensity area was not intentionally included in the CTV, and only edema in the expansion area was included. PTV1 and PTV2 were defined as CTV1 and CTV2 plus a 3 mm margin, respectively. For intensity-modulated radiotherapy (IMRT) planning, the dose prescribed for PTV2 was 54 Gy in 30 fractions, and the dose for PTV1 was 60 Gy in 30 fractions as a simultaneously integrated boost. Dose limits were set at 54 Gy for the optic nerves and optic chiasma and 59 Gy for the brainstem (10).

Concurrent chemotherapy with TMZ at 75 mg/m^2^/day began on the first day of radiotherapy and continued until the completion of radiation therapy. TMZ adjuvant chemotherapy (d1-5/28) was performed 4 weeks after radiotherapy. In the first cycle, the dose of TMZ was 150 mg/m^2^. If chemotherapy was well tolerated, it would increase to 200 mg/m^2^ from the second cycle forward. For patients with good general condition and drug tolerance, long-cycle TMZ adjuvant chemotherapy (more than six cycles of adjuvant chemotherapy) (11) was given. The dose was reduced or chemotherapy was suspended in the presence of disease progression or RTOG grade 3–4 toxicity.

### Follow Up and Evaluation of Recurrence

Patients were observed at all times during radiotherapy. After radiotherapy, regular outpatient follow-up visits and telephone follow-ups were performed until October 15, 2018. Enhanced cranial MRI was performed before radiotherapy, between the end of radiotherapy and the first adjuvant chemotherapy, and every 3 months after radiotherapy, or modified depending on the patient’s nervous system symptoms. MRI scans included contrast-enhanced T1-weighted and T2-weighted images, as well as sagittal and coronal contrast-enhanced T1-weighted images.

According to the Neuro-Oncology Working Group’s response assessment in neuro-oncology (RANO) evaluation criteria for treatment response (12), tumor recurrence is defined as an increase of 25% or more in the sum of the products of perpendicular diameters of enhancing lesions (compared with baseline minimum tumor measurements) or the emergence of new lesions. Pseudoprogression in patients with GBM usually occurs within 2–6 months after the end of concurrent radiochemotherapy and gradually decreases or even disappears after a few months (13). Therefore, the appearance of asymptomatic enlarged lesions around or within the primary tumor area, followed by natural disappearance or shrinkage, is considered to be pseudoprogression (14, 15). If the new lesions continue to increase in continuous MRI examination, this is considered to be tumor recurrence. Radiation necrosis typically involves the periventricular white matter within or adjacent to the radiation field due to its tenuous blood supply (15), but it can also occur in a contralesional and multifocal distribution (16). Internal enhancement patterns, described as “Swiss-cheese” or “soap-bubble,” have been shown to be more typical of radiation necrosis (16), as it has the presence of diffuse, “mesh-like enhancement” or peripheral enhancement with “feathery” margins (17, 18). On T2-weighted imaging, the central necrotic component will have a high signal, whereas the solid component will have a relatively lower signal (19). In patients with tumor progression, the time of recurrence was recorded when the first cranial MRI showed tumor progression. To judge tumor recurrence, at least one of the following four conditions was needed: 1. Multiple postoperative dynamic follow-ups of craniocerebral enhanced MRI-confirmed recurrence. 2. Comprehensive magnetic resonance perfusion imaging (PWI), magnetic resonance spectroscopy (MRS), diffusion-weighted imaging (DWI), and other examination methods combined with clinical symptoms. 3. recurrence was confirmed by pathology after reoperation. 4. Identification of tumor recurrence after multidisciplinary discussion in more difficult cases.

### Recurrence Pattern Identification

The contrast-enhanced T1-weighted images of the first follow-up and tumor recurrence were introduced into the Varian Eclipse radiotherapy planning system and fused with the original Sim-computed tomography (CT) images. The target area of the recurrent tumor was delineated, and the volume of the recurrent tumor was determined based on CT and MRI fusion images. The recurrent volumes of all patients were recorded by reviewers blinded to the original radiation volume to reduce the measurement bias caused by volume mapping and to describe the spatial relationship between recurrent tumor volume and the 60 Gy isodose line (IDL). Recurrence patterns were classified as follows (20–22): central recurrence, in-field recurrence, marginal recurrence, and distant recurrence, where more than 95%, 80–95%, 20–80%, and lower than 20% of the recurrence volume overlaps within 60 Gy IDL, respectively. Recurrence near the ventricle or cerebral cortex may be cerebrospinal fluid dissemination (CSF-d) and is separated from distant recurrence (9).

In all recurrent tumors, a virtual radiotherapy plan was developed according to the EORTC target delineation guidelines (5). We compared them with the actual radiotherapy plans. The principle of EORTC delineation is that one target volume is irradiated for 60 Gy/30 Fx. and GTV includes contrast-enhancing areas in the T1-weighted MRI sequences and operative cavity, but it does not include the T2/FLAIR hyperintensity area outside of the GTV. CTV is GTV expansion 2 cm for skull, ventricle, cerebral falx, tentorial cerebellum, optic organ, brainstem, and other natural barrier areas to expand 0–0.5 cm. PTV is CTV external 0.3 cm.

### Follow Up

For follow-up of the cranial MRI, the following situations were regarded as loss of follow-up: irregular follow-up (the interval between the two follow-ups was more than 3 months); cranial MRI was examined in another hospital; or no cranial MRI was available at the time point of tumor recurrence. The follow-up time was calculated from the date of pathological diagnosis, and the endpoint of observation was the death of the patient or the termination of the study. The time of recurrence was from the date of pathological diagnosis to the time of the first MRI imaging to judge the progression of the tumor. Survival time was from the date of pathological diagnosis to the end of study or death.

### Statistical Analysis

IBM SPSS 23.0 and R 3.5.2 statistical software were used for statistical analysis. The survival data were described by the Kaplan-Meier method and compared with the log-rank test. The normality test was carried out by the Kolmogorov-Smirnov method. For comparison between groups, the chi-square (*X* ^2^) test was used for classification variables, t-test for continuity variables, and rank-sum test for rank data. A two-tailed P value of less than 0.05 was considered significant in this study.

## Results

### Characteristics of Clinical Data

**Figure 1:**
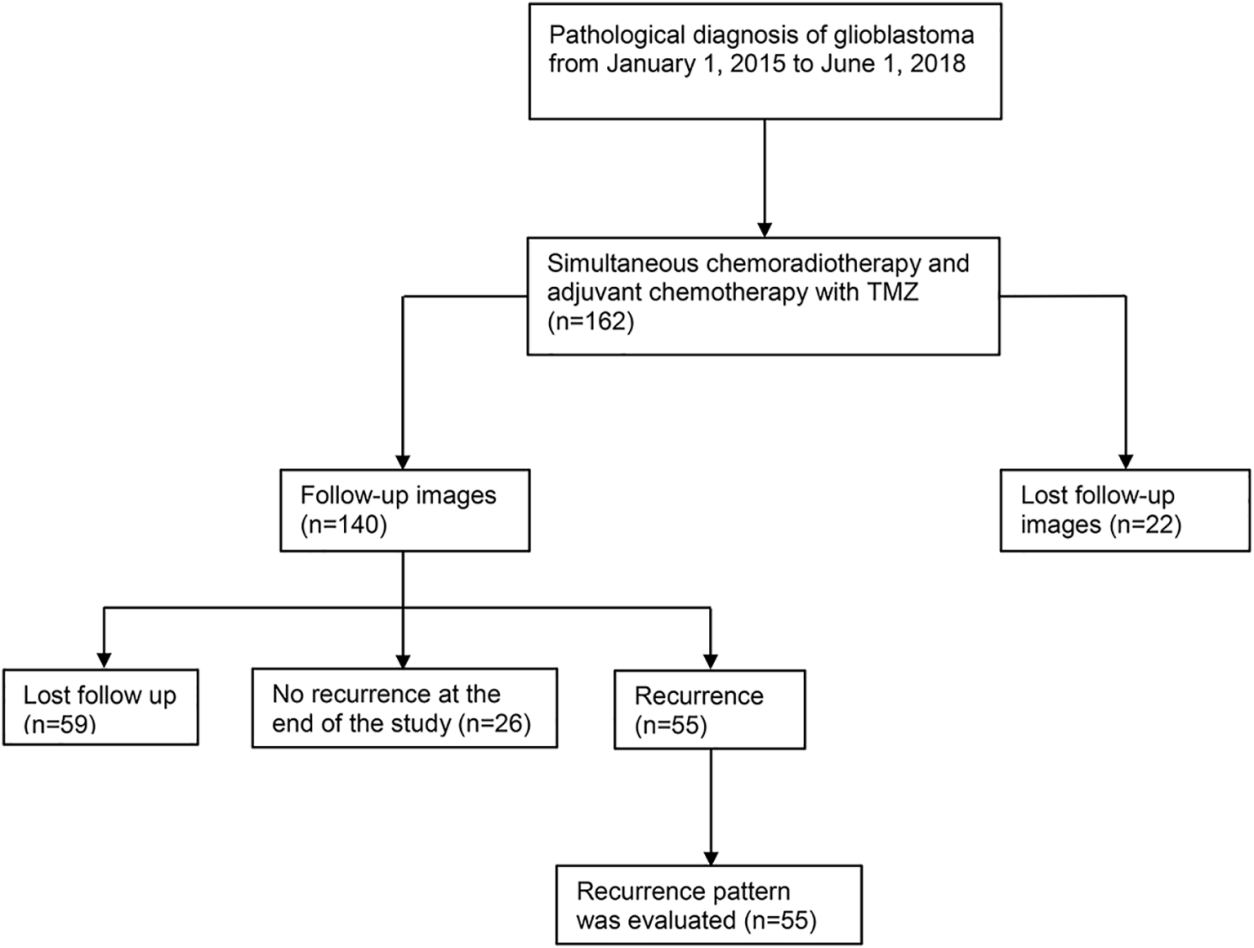
Flow chart of case selection. A total of 162 patients with pathologically confirmed glioblastoma were treated with postoperative concurrent radiochemotherapy and adjuvant chemotherapy. A total of 59 cases were lost to follow-up, 26 cases had no recurrence at the end of the study (October 15, 2018), and 55 cases recurred. Among the 55 recurrent patients, 13 cases were confirmed by reoperation and pathology, and the remaining 42 cases were diagnosed many times by dynamic MRI enhancement. In these patients, clinical symptoms were combined with PWI, MRS, DWI, and other examination items. Fifteen of the 42 patients presented diagnostic difficulties, and tumor recurrence was considered by multidisciplinary discussion. There were 2 cases with radiation necrosis and 6 with pseudoprogression out of 55 patients. TMZ, temozolomide; MRI, magnetic resonance imaging; PWI, magnetic resonance perfusion imaging; MRS, magnetic resonance spectroscopy; DWI, diffusion-weighted imaging.

From January 1, 2015, to June 1, 2018, we prospectively collected 162 newly pathologically confirmed patients with GBM and screened these cases. Fifty-five of these patients were included to retrospectively analyse the pattern of recurrence. **Figure 1** shows the flow chart of collecting cases. Among the 55 patients, there were 31 males and 24 females. The median age was 54 years (range: 21–80 years), and the median Karnofsky score was 90 (range: 50–90). A total of 24 patients underwent total tumor resection, 28 patients received subtotal resection, and three patients received biopsy only. Among the 55 patients with recurrence, 25 patients completed six cycles of adjuvant chemotherapy, of which 16 patients had more than seven cycles (median 12 cycles, range: 8–17 cycles). Fifty-nine patients were lost to follow-up at a median of 7.9 months. Among them, 27 patients completed six cycles of adjuvant chemotherapy, of which 12 patients had more than seven cycles (median 12 cycles, range: 7–26 cycles). The other 32 patients did not complete six cycles of adjuvant chemotherapy in our hospital. Eight patients refused to continue chemotherapy because of RTOG grade 3–4 toxicity, and seven patients returned to their local hospital to continue treatment. The chemotherapy cycle of seven cases was unknown. The clinical data of the patients are listed in **Table 1**.

**Table 1:** Clinical data characteristics of patients with GBM (n = 55).

Characteristics	n (%)
Gender	
male	31 (56.4%)
female	24 (43.6%)
Age	
Median (range)	54 (21–80)
<50	19 (34.5%)
≥50	36 (65.5%)
KPS	
Median (range)	90 (50–90)
<70	9 (16.4%)
≥70	46 (83.6%)
Mode of operation	
total resection	24 (43.6%)
subtotal resection	28 (51.0%)
biopsy	3 (5.4%)
Pathological type	
glioblastoma	55
Ki-67	
<5%	0 (0.0%)
5%-25%	13 (26.0%)
26%-50%	34 (68.0%)
>50%	3 (6.0%)
unknown	5
MGMT promoter status	
methylated	34 (72.3%)
unmethylated	13 (27.7%)
unknown	8
IDH	
Negative	42 (85.7%)
Positive	7 (14.3%)
unknown	6

GBM, glioblastoma; MGMT, O^6^-methylguanine-DNA methyltransferase; IDH, isocitrate dehydrogenase.

### Survival

**Figure 2:**
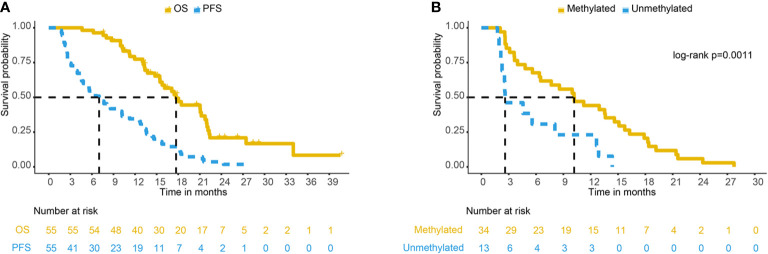
**(A)** PFS and OS curve of 55 GBM patients. **(B)** PFS comparison of different MGMT methylation statuses in 47 GBM patients. PFS, progression-free survival; OS, overall survival; GBM, glioblastoma; MGMT, O^6^-methylglucamine-DNA methyltransferase.

The median follow-up time was 15.8 months (range: 4.6–40.6 months). The median survival time of 55 patients with GBM was 17.7 months [95% confidence interval (CI): 15.5–21.9 months], and the 1- and 2-year OS rates were 77.3 and 19.6%, respectively. The median progression-free survival (PFS) was 7.0 months (95% CI: 4.8–11.2 months), and the 1- and 2-year PFS rates were 33.55 and 2.92%, respectively. **Figure 2A** shows the Kaplan-Meier survival curve of 55 patients with GBM.

### Recurrence Pattern

**Figure 3:**
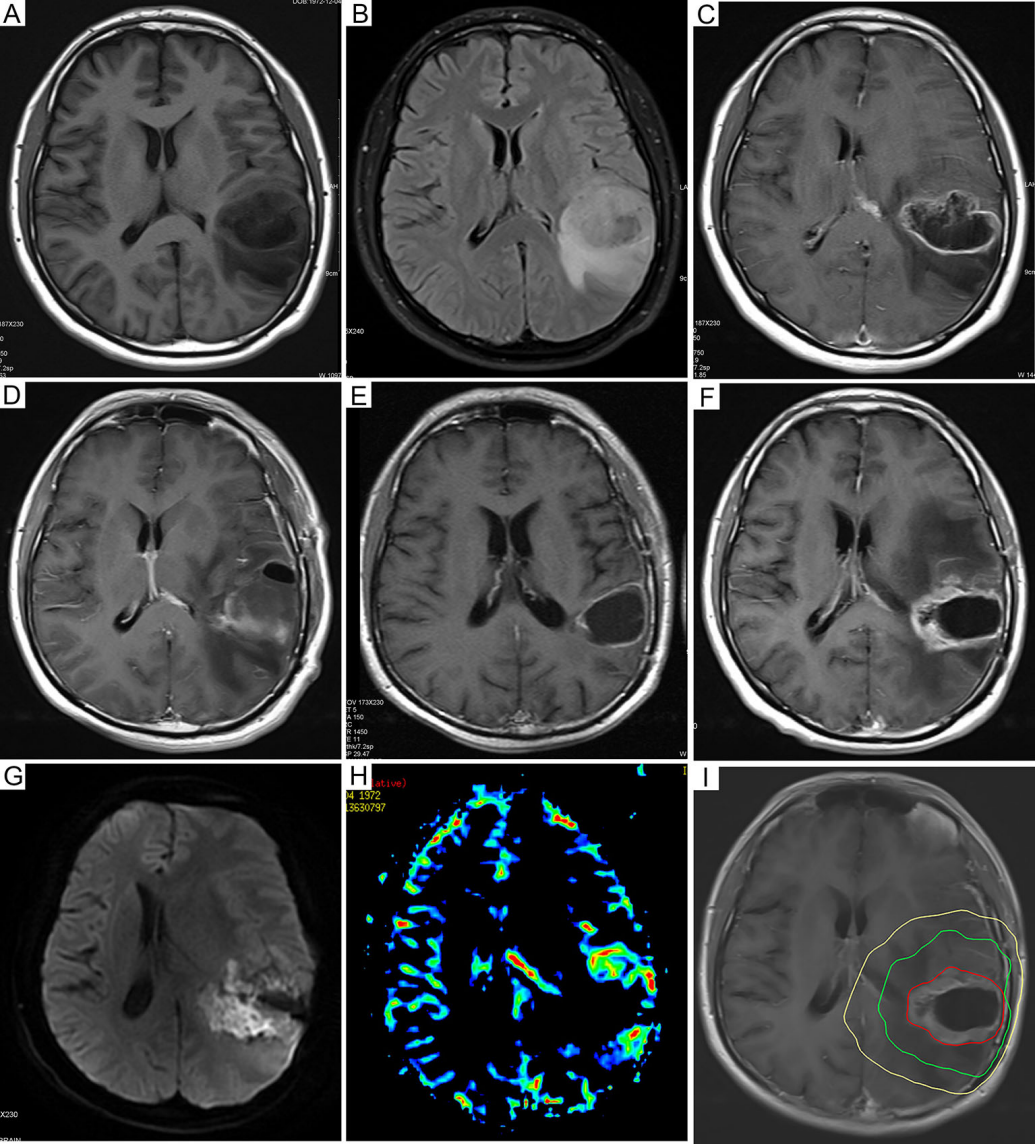
Dynamic follow-up cranial MRI and recurrence mode images of representative GBM patients. **(A)** Preoperative axial plain T1-weighted images and **(B)** axial fluid-attenuated inversion recovery images before resection. **(C)** Preoperative axial enhanced T1-weighted images showed that the tumor was in the left temporoparietal lobe. **(D)** Axial enhanced T1-weighted images 72 hours after resection. **(E)** The axial enhanced T1-weighted follow-up images 6 months after the operation showed new enhanced lesions at the level of the primary lesions. **(F)** Six months after resection, the axial enhanced T1-weighted follow-up images showed new enhanced lesions at the level of the primary lesions. **(G)** Further examination of DWI showed that local diffusion was limited, as well as high signal intensity. **(H)** In addition, magnetic resonance PWI showed nodular hyperperfusion around the lesion. The axial enhanced T1-weighted images of **(I)** were fused with the CT images of radiotherapy planning, indicating central recurrence. The yellow line, the green line, and the red line represent the isodose lines of 54 Gy, 60 Gy, and the volume of tumor recurrence, respectively. MRI, magnetic resonance imaging; GBM, glioblastoma; DWI, diffusion weighted imaging; PWI, perfusion weighted imaging.

Fifty-five patients could be used to analyse the pattern of recurrence. As shown in **Table 2**, 44 patients (80.0%) had central recurrences in the median 5.6 months (range: 1.6–24.3 months) after pathological confirmation, two patients (3.6%) had recurrences in the field at 7.3 and 12.6 months, respectively, and one patient (1.8%) had marginal recurrence at 18.4 months. Eleven patients (20.0%) developed distant recurrences in a median time of 14.6 months (range, 2.6–27.7 months), and three patients (5.5%) developed subependymal recurrences in a median time of 12.9 months (range: 10–21.6 months). Among them, five patients had multiple recurrence patterns. **Figure 3** shows a representative case of GBM outcome.

**Table 2:** Recurrence pattern and PFS in patients with GBM.

Recurrence pattern	n (%)	PFS median (range)	Recurrence within 6 months (%)	Recurrence within 12 months (%)	Recurrence within 18 months (%)
Central	44 (80.0)	5.6 (1.6-24.3)	54.5	75.0	93.2
In-field	2 (3.6)	10.0 (7.3-12.6)	0.0	50.0	100.0
Marginal	1 (1.8)	18.4	0.0	0.0	0.0
Outside	11 (20.0)	14.6 (2.6-27.7)	18.2	27.3	72.7
Subependymal	3 (5.5)	12.9 (10.0-21.6)	0.0	33.3	66.7

GBM, glioblastoma; PFS, progression-free survival.

### Comparison of Recurrence Pattern

Among the 55 recurrent patients, 47 patients had definite promoter methylation status, and eight patients’ methylation status was unknown. Of the 47 patients with definite promoter methylation status, 34 (72.3%) were methylated by the MGMT promoter, and 13 (27.7%) were not methylated by the MGMT promoter. The median survival time of patients with promoter methylation was 21.0 months and that of unmethylated patients was 17.7 months (log-rank p = 0.57). The median PFS times of patients with promoter methylation were 10.2 and 2.6 months (log-rank p = 0.0011), respectively (**Figure 2B**). In patients with MGMT methylation, there were 24 cases of central recurrence, one case of marginal recurrence, 11 cases of distant recurrence, and three cases of subependymal recurrence (4 of 34 cases had multiple recurrence patterns at the same time). In those with unmethylated tumors, there were 12 cases of central recurrence and one case of field recurrence, and no cases developed marginal recurrence, distant recurrence, or subependymal recurrence. There was a significant difference in the recurrence patterns in patients with promoter methylation versus unmethylation (Mann-Whitney U test, P = 0.026). The analysis of MGMT promoter methylation status showed that patients with MGMT promoter methylation were more likely to have distant recurrence (32.35 vs. 0%).

**Figure 4:**
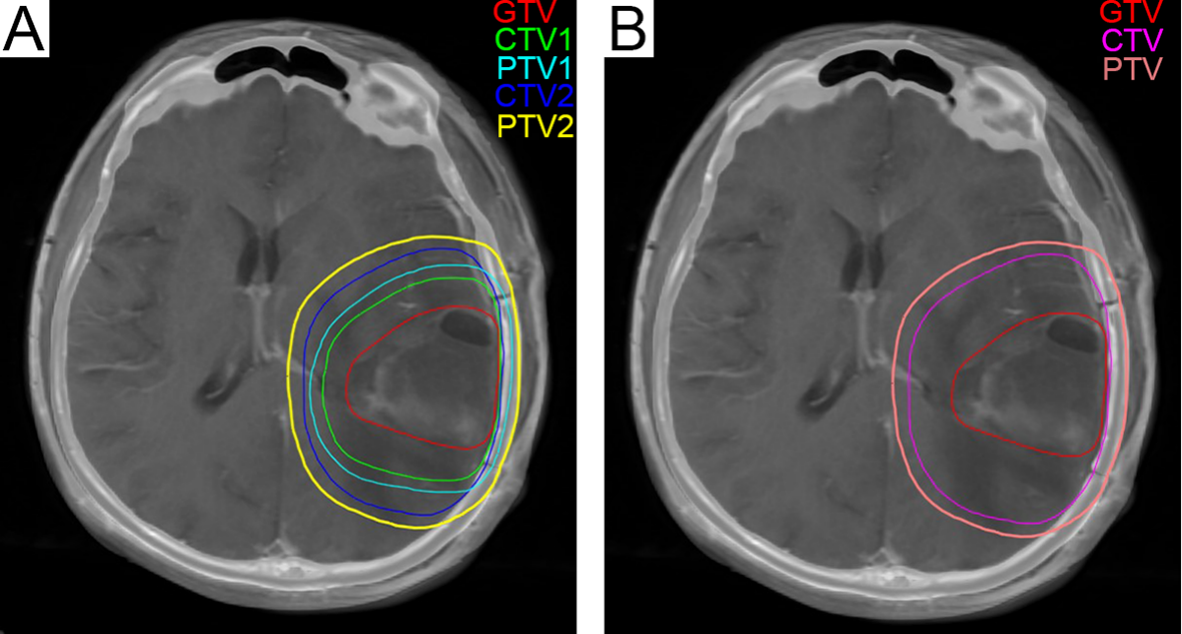
**(A)** The green line represents CTV1 in the radiation treatment planning in our center, and 204.6 cm^3^ was exposed to a dose of 60 Gy. **(B)** The fuchsia contour represents the CTV in the EORTC radiotherapy plan, and 347.1 cm^3^ was exposed to a dose of 60 Gy. CTV, Clinical Tumor Volume; EORTC, European Organization for Research and Treatment of Cancer.

The recurrence pattern of our regimen was similar to that of EORTC (8) and RTOG (23) (P = 0.882) (**Table 3**). Radiation planning did not intend to include edema, so the median brain volume of high-dose radiation fields was smaller than that of EORTC (P = 0.000) (**Table 4**). The comparison of recurrence patterns between our radiotherapy plan and the EORTC virtual plan is shown in **Table 5**. **Figure 4** shows a representative example of target volume contouring according to our plan and the EORTC plan. According to the principle of EORTC target delineation, the 1-year and 2-year survival rates of patients in clinical studies were 66 and 24%, respectively (8). According to RTOG target delineation, the 1-year and 2-year survival rates of patients in clinical studies were 63.8 and 31.8%, respectively (23). Similarly, the 1-year and 2-year survival rates in our institution were 77.3 and 19.6%, respectively. There was no significant difference in the 1-year and 2-year survival rates among the three groups (Pearson *X^2^* = 3.668 and 2.896, P = 0.160 and 0.235).

**Table 3:** Comparison of tumor recurrence patterns with different target delineation methods.

Recurrence volume within radiation field	Our plan n (%)	EORTC n (%)	RTOG n (%)
Central	44 (80.0%)	79 (75.3%)	55 (77.5%)
In-field	2 (3.6%)	6 (5.7%)	0 (0%)
Marginal	1 (1.8%)	6 (5.7%)	0 (0%)
Outside	11 (20.0%)	6 (5.7%)	6 (8.4%)
Subependymal	3 (5.5%)	8 (7.6%)	10 (14.1%)

Kruskal-Wallis H test (rank test of multiple independent samples), P = 0.882. The marginal recurrence rates of the three groups were tested by the Monte Carlo Fisher X^2^ test. X^2^ = 4.573, P = 0.077. The recurrence pattern of our regimen was similar to that of EORTC and RTOG. EORTC, European Organisation for Research and Treatment of Cancer; RTOG, Radiation Therapy Oncology Group.

**Table 4:** Comparison of brain volume exposed to radiation between our radiotherapy and EORTC virtual plan.

	Our plan	EORTC virtual plan
Dose received (Gy)	60	60
Median volume (cm^3^)	160.58	285.39^*^
Range	7.7–757.9	66.2–867.3
Standard deviation (SD)	129.38	146.95

Paired t-test *P = 0.000 between our plan and EORTC plan at a dose of 60 Gy. EORTC, European Organisation for Research and Treatment of Cancer.

**Table 5:** Comparison of recurrence patterns between our radiotherapy plan and EORTC virtual plan.

Recurrence volume within radiation field	Our plan n (%)	EORTC virtual plan n (%)
Central	44 (80.0%)	45 (81.8%)
In-field	2 (3.6%)	4 (7.3%)
Marginal	1 (1.8%)	1 (1.8%)
Outside	11 (20.0%)	8 (14.5%)
Subependymal	3 (5.5%)	3 (5.5%)

For the nonparametric test of the two related samples, P = 1.000. Both samples had marginal recurrence rates of 1.8%. The recurrence pattern between our plan and the EORTC virtual plan was similar. EORTC, European Organisation for Research and Treatment of Cancer; RTOG, Radiation Therapy Oncology Group.

## Discussion

The GTV used in this study does not intentionally include the T2/FLAIR hyperintensity area. After radiotherapy with concurrent and adjuvant chemotherapy based on TMZ, we studied the mode of tumor recurrence in patients with GBM and followed up on their PFS and OS. The results in our study showed that most local recurrences were located in the center of the field, and only one patient (1.8%) developed marginal recurrence. Compared with the EORTC and RTOG regimens (8, 23), the recurrence patterns among the three regimens were similar (P = 0.882). The analysis of MGMT promoter methylation status showed that patients with MGMT promoter methylation were more likely to have distant recurrence (32.35 vs. 0%). The 1-year and 2-year survival rates of the patients were 77.3 and 19.6%, respectively, and the survival outcome was similar to the results of other clinical studies (8, 23) (P = 0.160 and 0.235).

Radiotherapy is an important component of GBM treatment. To date, there is still no consensus on the radiotherapy plan for GBM. At present, the protocols outlined by RTOG (4) and EORTC (5) are commonly used (**Table 6**). The target areas of the RTOG guidelines (including RTOG 83-02, 86-12, and 97-10) emphasize the presence of edema (4, 24–26), and the irradiation volume is relatively large. Kruser et al. agreed with the opinion of the RTOG/NRG guidelines based on autopsy and MRI guided stereotactic biopsy, tumor cells were found in the T2 edema region (7). Tseng et al.’s results support the need to develop individualized irradiation strategies for glioblastomas according to extensive preoperative edema (EPE) and synchronous subventricular zone and corpus callosum (sSVZCC) (27). However, Chang et al. (20) and Minniti et al. (8) reported that target delineation did not need to intentionally include the edema area, and it did not change the failure mode of GBM patients. Wee et al. assessed the differences in the target volume of newly diagnosed glioblastoma drawn by 15 different radiotherapy institutions in Korea, and the studies showed that the centrally failing pattern of GBM does not change even with reduced margins (28). A series of previous studies have demonstrated that most recurrences occur within 2 cm of the edge of the tumor, indicating that there is little relationship between tumor recurrence and edema (29–35). Therefore, a small radiotherapy field may be more appropriate than a large radiotherapy field, especially in patients with large edema areas. Moreover, according to our plan, the median brain volume of high-dose irradiation was significantly lower than that of the EORTC plan (P = 0.000). Our study showed that radiotherapy plans not intending to include the adjacent T2/FLAIR hyperintensity area did not increase the risk of marginal recurrence.

**Table 6:** Comparison of the target definition and delineation principles among our study, EORTC, and RTOG.

Group	Our plan*	EORTC	RTOG
Phases	60Gy	60Gy	46+14 = 60Gy
GTV1	T1+cavity (post-op)	T1+cavity (post-op)	T1+cavity (post-op)+T2 FLAIR
GTV2	None	None	T1+cavity(post-op)
CTV1	GTV+1 cm	GTV+2 cm	GTV1+2 cm
CTV2	GTV+2 cm	none	GTV2+2 cm
PTV1	CTV1+3 mm	3–5 mm	CTV1+3-5 mm
PTV2	CTV2+3 mm	None	CTV2+3-5 mm

*We defined the GTV as the postoperative T1-weighted MRI enhancement area and postoperative residual cavity. CTV1 and CTV2 were defined as GTVs with 1 and 2 cm expansion, respectively. PTV1 and PTV2 were defined as CTV1 and CTV2 plus a 3 mm margin, respectively. For IMRT planning, the dose prescribed for PTV2 was 54 Gy in 30 fractions, and the dose for PTV1 was 60 Gy in 30 fractions as a simultaneously integrated boost. EORTC, European Organisation for Research and Treatment of Cancer; RTOG, Radiation Therapy Oncology Group; GTV, gross tumor volume; CTV, clinical target volumes; PTV, planning target volume; post-op, postoperative; FLAIR, Fluid Attenuated Inversion Recovery Image; IMRT, intensity-modulated radiotherapy.

In this study, analysis of MGMT promoter methylation status showed that the recurrence patterns were different (P = 0.026). Patients with MGMT promoter methylation were more likely to have distant recurrence (32.35%). Minniti et al. (8) reported recurrence patterns after concurrent radiotherapy and chemotherapy in 105 glioblastoma patients; 64 and 31% of MGMT-methylated patients had central/in-field and distant recurrence, respectively, in contrast to 91% and 5.4% of unmethylated patients. In the study of Brandes et al. (36), 95 patients with GBM received radiotherapy and TMZ chemotherapy. Among them, 42% of the patients with MGMT promoter methylation had distant metastasis.

However, our research has several limitations: 1. A small sample size and only recurrent patients with complete follow-up data were included. Selection bias might have existed. 2. Patients in reported studies had different prognostic variables, so survival could not be compared across studies.

With the progress of treatment and prolonged survival, more patients undergoing radiotherapy will develop late radiation neurotoxicity, including cognitive decline and radiation brain necrosis, which will dramatically decrease the quality of life of patients (20). The degree of radiation neurotoxicity is related to the volume of normal brain tissue exposure. The smaller the radiation exposure volume, the fewer radiation-related adverse reactions will occur (20). Therefore, it is worth exploring to determine a better radiotherapy volume. In our study, there were only two cases of radioactive brain necrosis (3.64%), which was relatively low compared to 5–50%, which is reported in previous literature (15, 37). Our study showed that reducing the irradiation volume did not increase the risk of marginal recurrence.

In summary, delineating the enhancing area of T1-weighted MRI and the resection cavity as GTV is reasonable for GBM patients receiving chemoradiotherapy and adjuvant chemotherapy with TMZ. Compared with the EORTC scheme, target delineation that excludes the adjacent T2/FLAIR hyperintensity area reduces the brain volume exposed to high-dose radiation (P = 0.000) with a lower marginal recurrence rate (1.8%). In addition, there was no significant difference in the 1-year and 2-year survival rates, and reduced radiation volume might theoretically reduce adverse reaction rates. Therefore, it is worthwhile to conduct a clinical trial investigating the feasibility of intentionally not including the T2/FLAIR hyperintensity region outside of the GTV.

## Data Availability Statement

The raw data supporting the conclusions of this article will be made available by the authors, without undue reservation.

## Ethics Statement

The Ethics Committee of the Second Affiliated Hospital of Medical College of Zhejiang University approved retrospective analysis of patient data. The patients/participants provided their written informed consent to participate in this study.

## Author Contributions

LZ, Z-RZ, QY, and QW conceived and designed the study and drafted the manuscript. MS and YY collected, analyzed, and interpreted the data. XZ and CL participated in revising the manuscript. All authors contributed to the article and approved the submitted version.

## Funding

This work was funded by the Chinese Society of Neuro-oncology, CACA grant (CSNO-2014-MSD05) and the Taizhou Science and Technology Program (No. 20ywb138).

## Conflict of Interest

The authors declare that the research was conducted in the absence of any commercial or financial relationships that could be construed as a potential conflict of interest.
